# Trends in the use of implantable cardioverter‐defibrillator and cardiac resynchronization therapy device in advancing age: Analysis of the Japan cardiac device treatment registry database

**DOI:** 10.1002/joa3.12377

**Published:** 2020-06-08

**Authors:** Hisashi Yokoshiki, Akihiko Shimizu, Takeshi Mitsuhashi, Kohei Ishibashi, Tomoyuki Kabutoya, Yasuhiro Yoshiga, Ritsuko Kohno, Haruhiko Abe, Akihiko Nogami, Members of the Implantable Cardioverter-Defibrillator (ICD) Committee of the Japanese Heart Rhythm Society

**Affiliations:** ^1^ Department of Cardiovascular Medicine Sapporo City General Hospital Sapporo Japan; ^2^ UBE Kohsan Central Hospital Ube Japan; ^3^ Cardiovascular Medicine Jichi Medical University Saitama Medical Center Saitama Japan; ^4^ Department of Cardiovascular Medicine National Cerebral and Cardiovascular Center Suita Japan; ^5^ Division of Cardiovascular Medicine Department of Medicine Jichi Medical University School of Medicine Shimotsuke Japan; ^6^ Division of Cardiology Department of Medicine and Clinical Science Yamaguchi University Graduate School of Medicine Yamaguchi Japan; ^7^ Department of Heart Rhythm Management University of Occupational & Environmental Health Kitakyushu Japan; ^8^ Cardiovascular Division Faculty of Medicine University of Tsukuba Tsukuba Japan

**Keywords:** advancing age, cardiac resynchronization therapy with a defibrillator (CRT‐D), cardiac resynchronization therapy with a pacemaker (CRT‐P), implantable cardioverter‐defibrillator (ICD), primary prevention

## Abstract

**Background:**

Trends of de novo implantation of cardiac implantable electronic devices (CIEDs) including implantable cardioverter‐defibrillator (ICD) and cardiac resynchronization therapy with a defibrillator (CRT‐D) or pacemaker (CRT‐P) in advancing age are unknown.

**Methods:**

Analysis of data from the Japan cardiac device treatment registry (JCDTR) with an implantation date between January 2006 and December 2016 was performed focusing on advancing age of ≧75 years.

**Results:**

The cohort included 17 564 ICD, 9470 CRT‐D and 1087 CRT‐P recipients for de novo implantation. The rate of patients ≧75 years of age increased from 17.1% to 20.5% in ICD implantation (*P* = .052), from 19.7% to 30.0% in CRT‐D implantation (*P* < .0001), and from 40.0% to 64.0% in CRT‐P implantation (*P* = .17). There was an apparent increase in the percentage of nonischemic patients aged ≧75 years receiving ICD (10.9% in 2006 to 16.4% in 2016, *P* = .0008) and CRT‐D (17.1% in 2006 to 27.8% in 2016, *P* = .0001). The implantation for primary prevention ICD (*P* = .059) and CRT‐D (*P* = .012) was also associated with a temporal increase in the percentage of patients aged ≧75 years.

**Conclusions:**

Proportion of patients ≧75 years of age for de novo CIED implantation gradually increased from 2006 to 2016, presumably because of the growing number of nonischemic cardiomyopathy and heart failure patients requiring primary prevention of sudden cardiac death.

## Graphical abstract

Proportion of patients ≧75 years of age for de novo CIED implantation gradually increased from 2006 to 2016, presumably due to the growing number of non‐ischemic cardiomyopathy and heart failure patients requiring primary prevention of sudden cardiac death.

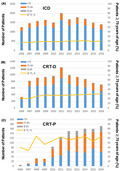

## 1 INTRODUCTION

Implantable cardioverter‐defibrillator (ICD) therapy has been proved to be effective for primary prevention of sudden cardiac death in patients with symptomatic heart failure with reduced ejection fraction (HFrEF),^1^, ^2^, ^3^ and its use is prevailing as one of the standard therapies in combination with guideline‐directed medical therapy. Age of patients enrolled in randomized controlled trials was 65 ± 10 years old (means ± SD) in the Multicenter Automatic Defibrillator Implantation Trial II (MADIT II),^3^ 60 years old (median) in the Sudden Cardiac Death in Heart Failure Trial (SCD‐HeFT)^1^ and around 67 years old (median) in the Comparison of Medical Therapy, Pacing and Defibrillation in Heart Failure (COMPANION) study.^2^ On the other hand, the Amiodarone Trialists MetAnalysis (ATMA) investigators demonstrated that sudden death to all‐cause death ratio decreased with age, which was 51% in those age <50 years and 26% in those after age 80 years.^4^ Moreover, in nonischemic patients with HFrEF, the association between reduced all‐cause mortality and ICD implantation became no significant with increasing age, and an optimal age cutoff for ICD implantation was present at ≦70 years.^5^

The United States (US) trends demonstrated that CRT‐P use decreased progressively from 2002 (28.8% of all CRTs) to 2010 (15.2% of all CRTs) and that the percentage of CRTs (CRT‐D and CRT‐P) implanted in patients aged ≧85 years increased from 4.9% in 2003 to 8% in 2010.^6^ In CRT recipients without a prior history of sustained ventricular arrhythmias, advancing age was significantly associated with the choice of CRT‐P over CRT‐D in Japan, resulting that the mean age of those receiving CRT‐P was 75 years old.^7^ This clinical practice could be reasonable because a defibrillator backup had subtle or no survival benefit in symptomatic heart failure patients requiring a CRT device in randomized controlled studies.^2^, ^8^, ^9^ However, Japanese trends in the age‐stratified use of ICD and CRT device have not been evaluated.

This study was aimed at examining temporal trends of cardiac implantable electronic devices (CIEDs), including ICD/CRT‐D/CRT‐P, implantation by analyzing the Japan Cardiac Device Treatment Registry (JCDTR) database and to explore the hypothesis that there is an increasing number of CIEDs implanted in older patients aged ≧75 years over a decade in the recent aging society in Japan.

## 2 METHODS

### 2.1 Study population

The JCDTR was established in 2006 by the Japanese Heart Rhythm Society (JHRS) for a survey of actual conditions in patients undergoing de novo implantation of CIEDs including ICD/CRT‐D/CRT‐P.^10^, ^11^, ^12^ A new system, called New JCDTR, started on January 2019, in which data of patients at the implantation date after January 2018 are encouraged to register (https://membnew.jhrs.or.jp/newjcdtr/ accessed on March 1, 2020). The protocol for this research project has been approved by a suitably constituted Ethics Committee of each institution and it conforms to the provisions of the Declaration of Helsinki. This study analyzed the data of implantation date from 2006 to 2016 according to age of patients.

**FIGURE 1:**
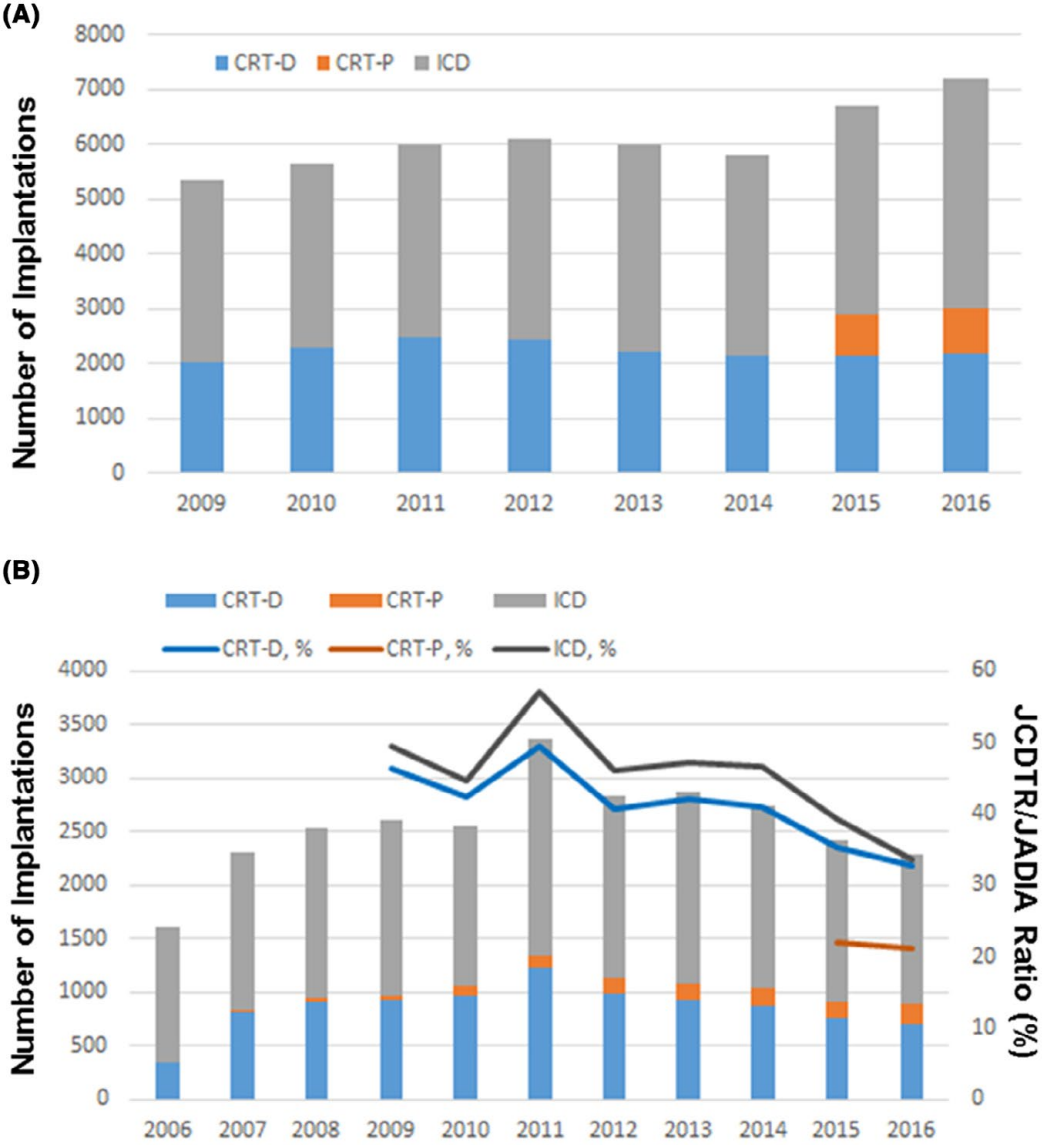
Japanese trends in de novo CIEDs implantation. The number of new implantations of ICD (gray bar)/CRT‐D (blue bar)/CRT‐P (orange bar) in each year is shown based on the data from (A) Japan Arrhythmia Device Industry Association (JADIA) and (B) Japan cardiac device treatment registry (JCDTR). Percentage of registration of the JCDTR to that of JADIA (JCDTR/JADIA ratio) is given as line graphs (B).

The Japan Arrhythmia Device Industry Association (JADIA) reports the annual number of de novo implantations of ICD and CRT‐D from 2009 and that of CRT‐P from 2015 (https://www.jadia.or.jp; Figure 1A). This study also evaluated the ratio of our registration to that reported in the JADIA (JCDTR/JADIA ratio).

### 2.2 Statistical analysis

All data are expressed as mean ± SD. Simple between‐group analysis was conducted using Student's *t* test. Multiple comparisons were assessed by ANOVA with the post hoc analysis using a Bonferroni test when necessary. Categorical variables were compared using the Chi‐square test. Differences with *P* < .05 were considered significant. Statview version 5.0 for Windows (SAS Institute Inc.) or R software ver.3.2.3 (https://www.r‐project.org/) was used for all statistical analyses.

## 3 RESULTS

### 3.1 Study cohorts

The JCDTR database constituted data of 28 121 patients who underwent de novo implantations of ICD, CRT‐D or CRT‐P from January 2006 to December 2016 (extracted on 29 September 2018). To be more specific, 17 564 ICD recipients, 9470 CRT‐D recipients and 1087 CRT‐P recipients were included for the evaluation (Table 1). With regard to age, gender, left ventricular ejection fraction (LVEF) and New York Heart Association (NYHA) class, there were significant differences among the three groups. The mean age of CRT‐P recipients was 74.3 years old, which was higher than 60.8 years old of ICD recipients (*P* < .0001) and 66.9 years old of CRT‐D recipients (*P* < .0001). The rate of ischemic heart disease was 36.4% in ICD recipients, which was highest among the three groups (*P* < .0001). The indication for defibrillation therapy was primary prevention in 66.7% of CRT‐D recipients and in 25.6% of ICD recipients (*P* < .0001; Table 1). The rate of CRT (CRT‐D and CRT‐P) recipients without a prior history of sustained ventricular arrhythmias (ie, primary prevention of sudden cardiac death) was 69.8% (not shown in Table 1). The number of registrations was maximum in 2011 which included 2007 patients with ICD, 1226 patients with CRT‐D and 124 patients with CRT‐P (Figure 1B).

**TABLE 1:** Characteristics of patients undergoing CIEDs implantation, stratified by device type

	ICD	CRT‐D	CRT‐P	*P* value
Number of patients	17564	9470	1087	
Age (y)	60.8 ± 15.4	66.9 ± 11.2	74.3 ± 11.1	<.0001
Male	13745 (78.2)	7172 (75.7)	671 (61.7)	<.0001
Underlying heart disease				<.0001
Ischemic	6394 (36.4)	2959 (31.2)	280 (25.8)	
Nonischemic	111170 (63.6)	6511 (68.8)	807 (74.2)	
Primary prevention^a^	4502 (25.6)	6317 (66.7)	1048 (96.4)	
LVEF (%)	50.0 ± 17.1	27.6 ± 9.2	32.6 ± 11.3	<.0001
NYHA class				<.0001
I	9822 (55.9)	344 (3.6)	35 (3.2)	
II	5742 (32.7)	2370 (25.0)	259 (23.8)	
III	1735 (9.9)	5648 (59.7)	705 (64.9)	
IV	265 (1.5)	1108 (11.7)	88 (8.1)	

Note

Values are means ± SD, or number (%).

Abbreviation: ICD, implantable cardioverter‐defibrillator; LVEF, left ventricular ejection fraction; NYHA, New York Heart Association.

^a^ Patients without a prior history of sustained ventricular arrhythmias are defined as primary prevention.

**FIGURE 2:**
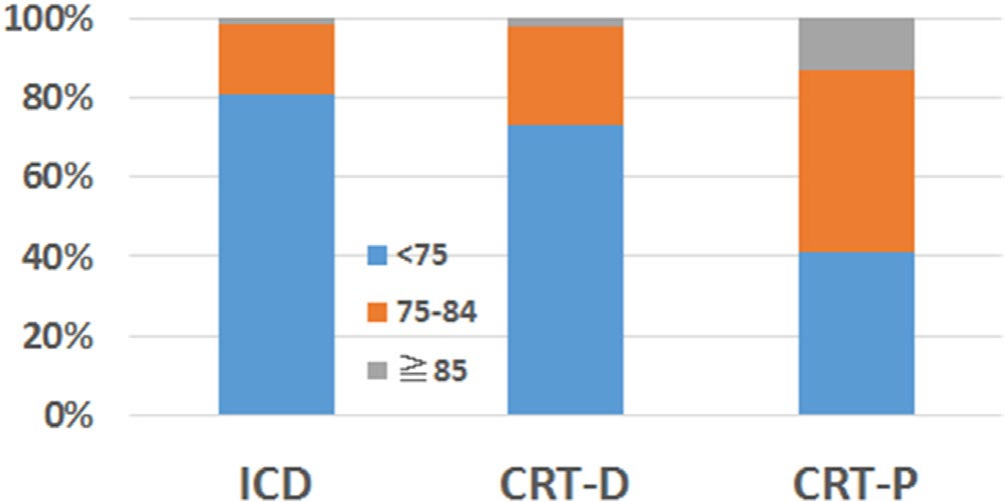
Proportion of different age groups in CIED implantation. Groups of patients aged <75 y (blue bar), aged 75‐84 y (orange bar) and aged ≧85 y (gray bar) are shown as the percentages for ICD, CRT‐D and CRT‐P implantation. The age distribution was significantly different among CIED implantations (*P* < .0001).

Proportion of patients aged ≧75 years and those ≧85 years was 19.3% and 1.7% in all ICD implantations, 27.0% and 2.0% in all CRT‐D implantations, and 59.0% and 13.0% in all CRT‐P implantations (Figure 2). The distribution of advancing age for these CIEDs implantation was significantly different (*P* < .0001).

### 3.2 Age‐stratified ICD/CRT‐D/CRT‐P implant trends

#### 3.2.1 Overall

**FIGURE 3:**
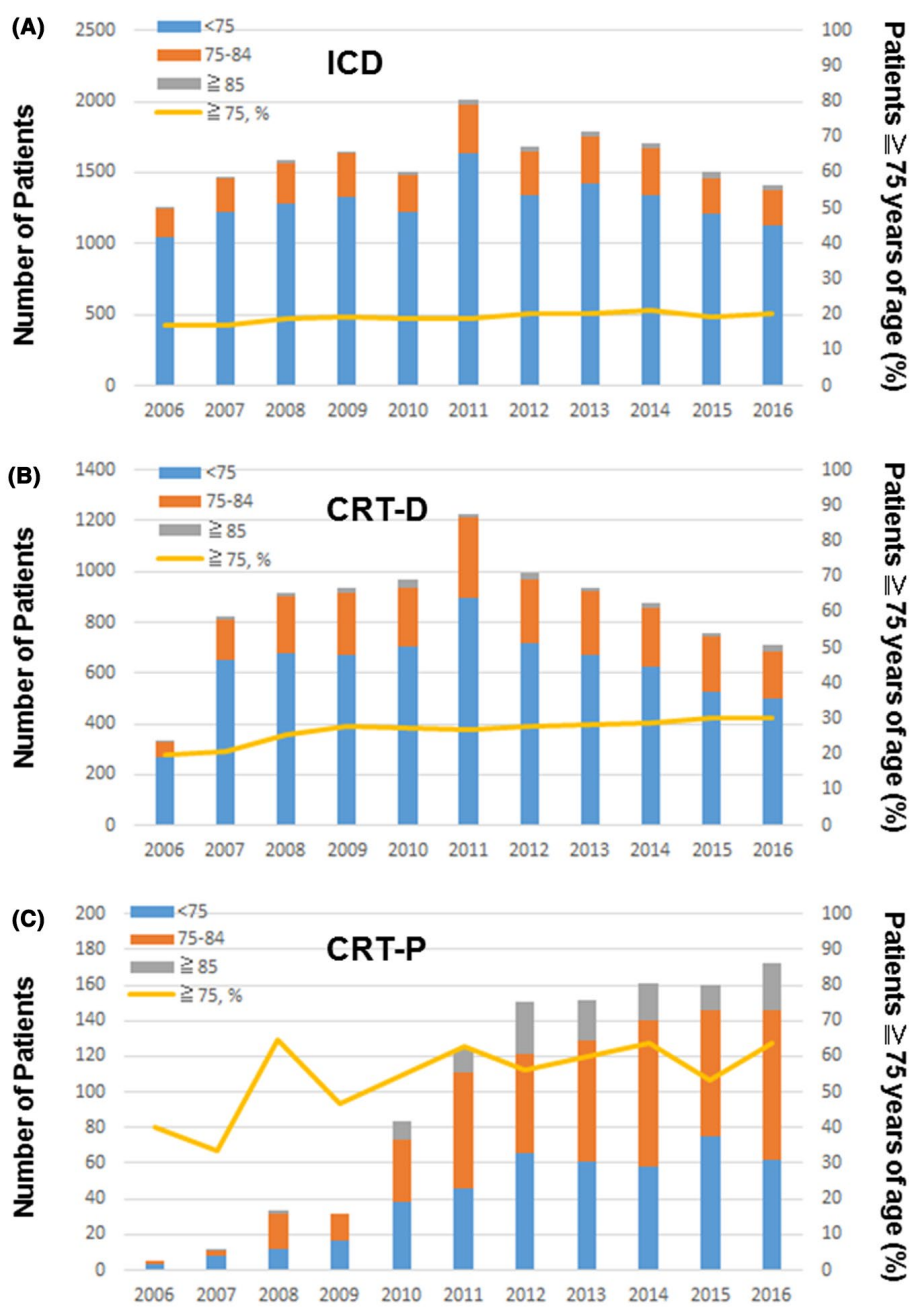
Age‐stratified CIEDs implant trends. Implantation trends in the patient groups, aged <75 y (blue bar), aged 75‐84 y (orange bar) and aged ≧85 y (gray bar), and the percentage of patients aged ≧75 y (yellow line) is given for ICD (A), CRT‐D (B) and CRT‐P (C) implantation. The percentage of patients aged ≧75 y increased significantly in CRT‐D implantation (*P* < .0001). The increase was marginal in ICD implantation (*P* = .052) and CRT‐P implantation (*P* = .17).

In 2006, the percentage of ICD recipients and CRT‐P recipients ≧75 years of age was 17.1% and 40%. The rate increased to 20.5% for ICD recipients and 64.0% for CRT‐P recipients in 2016, with a marginal significance (*P* = .052 for ICD, *P* = .17 for CRT‐P; Figure 3A,C). There was a significant increase in the percentage of CRT‐D recipients ≧75 years of age from 19.7% in 2006 to 30.0% in 2016 (*P* < .0001). The percentage of CRT‐D recipients ≧85 years of age was 1.8% in 2006, and it increased to 3.5% in 2016 (*P* = .0018; Figure 3B). The percentage of all CRTs (CRT‐D and CRT‐P) implanted in patients aged ≧75 years and those aged ≧85 years was increased from 20% and 1.8% in 2006 to 36.7% and 5.8% in 2016, respectively (*P* < .0001 for the two age groups).

#### 3.2.2 Etiology: ischemic vs nonischemic

**FIGURE 4:**
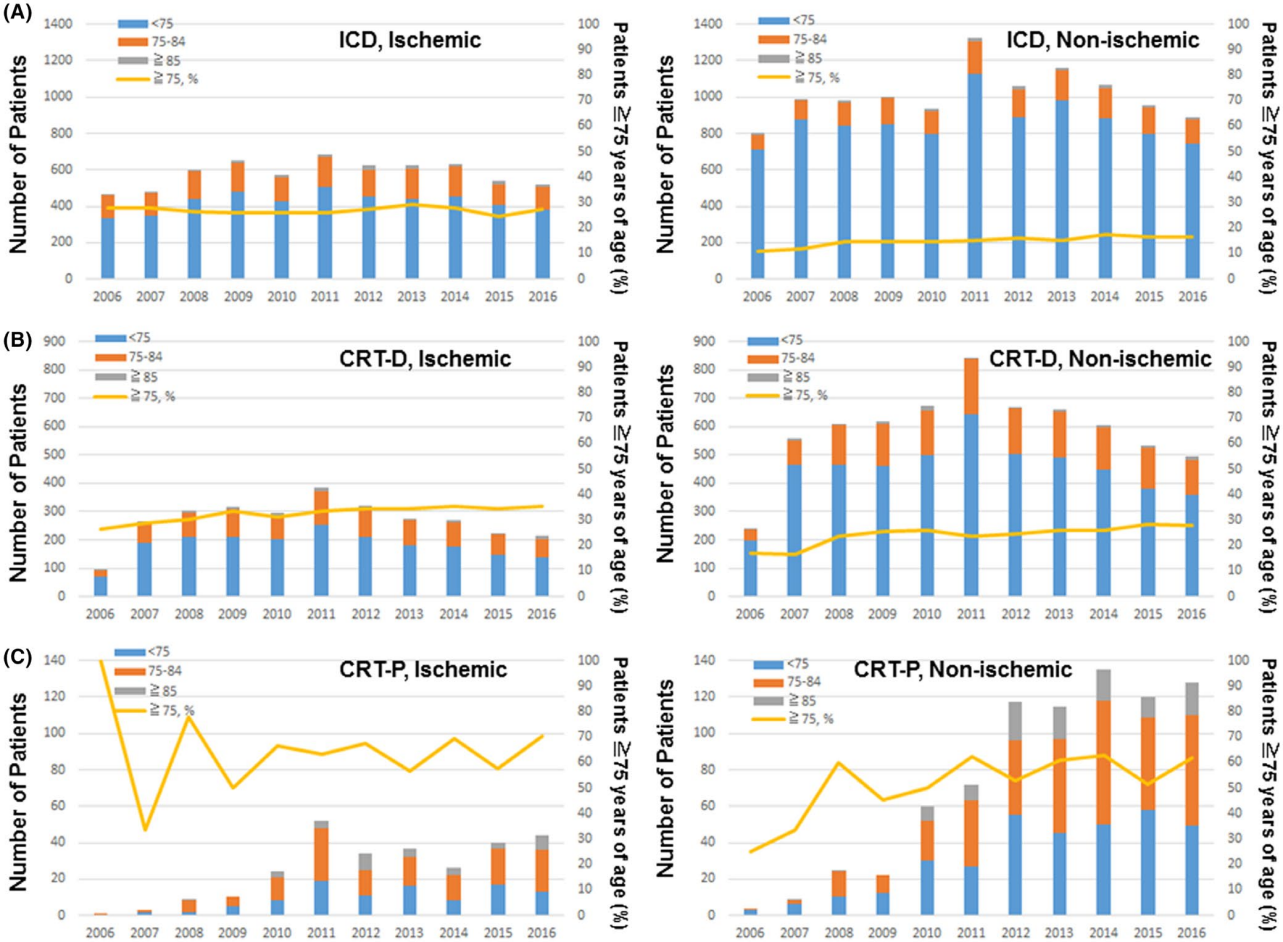
Age‐stratified CIED implant trends in ischemic and nonischemic etiologies. Implantation trends in three patient groups, aged <75 y (blue bar), aged 75‐84 y (orange bar) and aged ≧85 y (gray bar), and the percentage of patients aged ≧75 y (yellow line) are given for ischemic (left) and nonischemic (right) patients with ICD (A), CRT‐D (B) and CRT‐P (C) implantation. The percentage of patients aged ≧75 y increased significantly in nonischemic ICD (right panel in A, *P* = .0008) and CRT‐D (right panel in B, *P* = .0001) implantation. The increase was not significant in nonischemic CRT‐P implantation (right panel in C, *P* = .21). There was no statistical significant increase in age‐stratified implant trends for ischemic ICD, CRT‐D, and CRT‐P implantation (left panels in A, B and C).

With regard to the etiology of heart diseases, there was a significant increase in the percentage of nonischemic patients ≧75 years of age with ICD (10.9% in 2006 to 16.4% in 2016, *P* = .0008) and CRT‐D (17.1% in 2006 to 27.8% in 2016, *P* = .0001) implantations. There was an increasing trend in nonischemic CRT‐P recipients ≧75 years of age, but with no statistical significance (25% in 2006 to 61.7% in 2016, *P* = .22). The rate of ischemic patients ≧75 years of age changed with no statistical significance in ICD (28.0% in 2006 to 27.6% in 2016, *P* = .87), CRT‐D (26.3% in 2006 to 35.3% in 2016, *P* = .64) and CRT‐P (100% in 2006 to 70.5% in 2016) implantations (Figure 4).

#### 3.2.3 Indication: primary prevention vs secondary prevention

**FIGURE 5:**
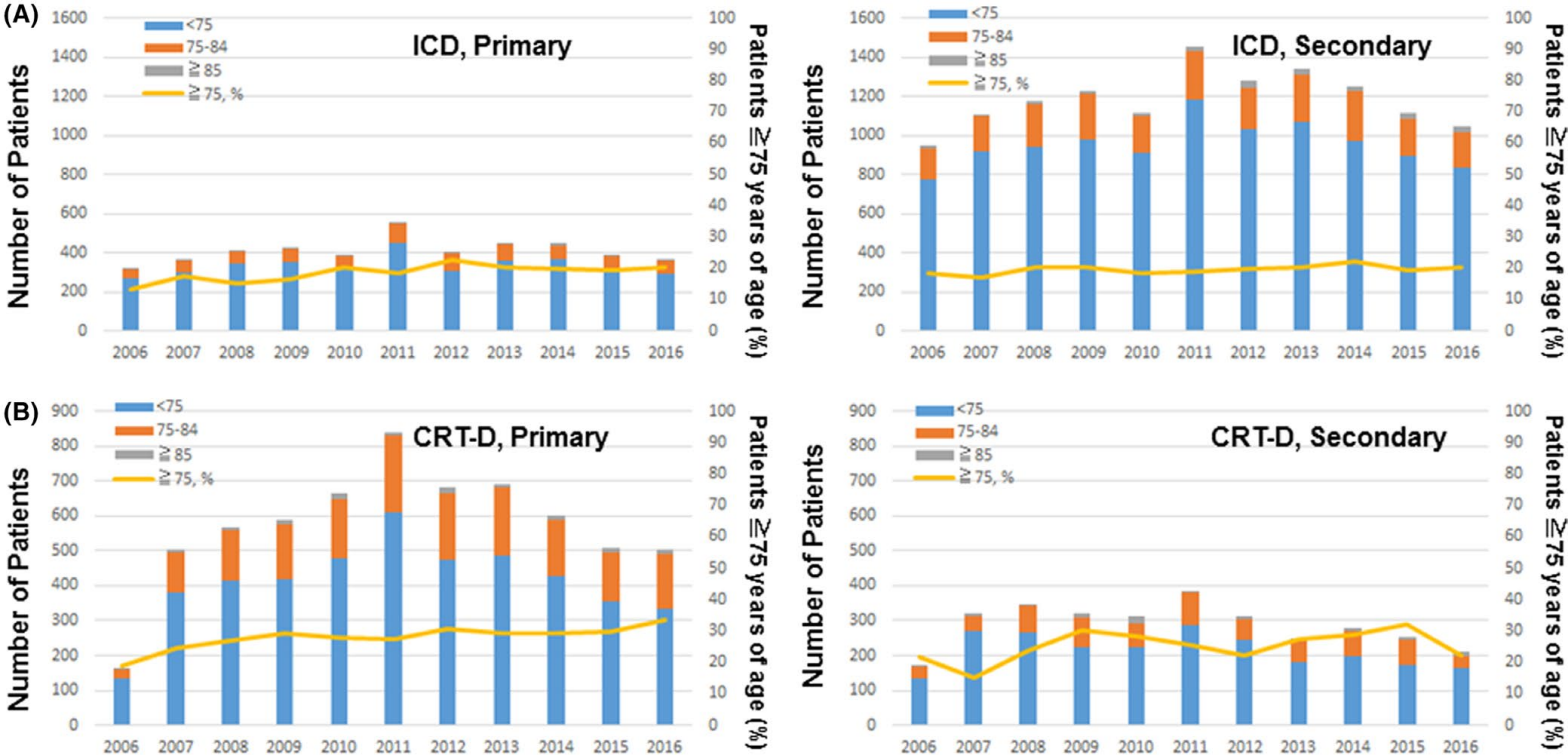
Age‐stratified CIED implant trends in primary prevention and secondary prevention for sudden cardiac death. Implantation trends in three patient groups, aged <75 y (blue bar), aged 75‐84 y (orange bar) and aged ≧85 y (gray bar), and the percentage of patients aged ≧75 y (yellow line) are given for primary prevention (left) and secondary prevention (right) ICD (A) and CRT‐D (B) implantation. The percentage of patients aged ≧75 y increased significantly only in primary prevention CRT‐D implantation (left panel in B, *P* = .012). The increase in the percentage of patients aged ≧75 y was marginal in primary prevention ICD implantation (left panel in A, *P* = .059).

The percentage of secondary prevention ICD recipients and CRT‐D recipients ≧75 years of age was 18.5% and 21.5% in 2006, and 20.5% and 22.0% in 2016. In contrast, there was a significant increase in the percentage of primary prevention CRT‐D recipients ≧75 years of age (18.8% in 2006 to 33.6% in 2016, *P* = .012). There was an increasing trend in the percentage of primary prevention ICD recipients ≧75 years of age (13.1% in 2006 to 20.4% in 2016 for ICD, *P* = .059; Figure 5).

## 4 DISCUSSION

The population of Japan decreased from 127.9 million in 2006 to 126.9 million in 2016, whereas the percentage of aged (≧75 years) people increased from 9.5% in 2006 to 13.3% in 2016 (https://www.stat.go.jp/data/jinsui/2016np). In accordance with this demographic change, this study demonstrated, with analyses of the JCDTR database, there has been a significant increase in the rate of de novo CIEDs implanted in patients with advancing age (ie, ≧75 years). Patients with nonischemic cardiomyopathy (but not ischemic cardiomyopathy) undergoing primary prevention CRT‐D and ICD significantly contributed to the increase in the implantation of the aged (≧75 years) population.

An observation that the percentage of all CRTs (CRT‐D and CRT‐P) implanted in patients aged ≧85 years increased from 1.8% in 2006 to 5.8% in 2016 was similar to the situation in the US,^6^ but the proportion was less than that reported in the US (6.7% in 2006, 8.0% in 2010).^6^ The rate of primary prevention CRT‐D implantation was 66.7% in this study (Table 1), which was lower than that of cohort studies in the US and Europe (72.4% in the US^13^ and 87% in Europe^14^). If symptomatic heart failure patients requiring a CRT device increase further and we opt to implant the device for primary prevention more frequently, the rate of CRT implantation in advancing age may become the same level as in the US.

In contrast to the higher prevalence of ischemic cardiomyopathy in the United States,^15^ about 70% of heart failure patients have nonischemic etiology in Japan (Table 1). In a subanalysis of the Cardiac Resynchronization – Heart Failure (CARE‐HF), patients with ischemic cardiomyopathy showed a higher incidence of the primary outcome and worse prognosis, as compared to nonischemic cardiomyopathy.^16^ Therefore, the rate of ischemic patients of advancing age who have an indication for CRT‐D may not be increasing to the same extent of nonischemic patients. In addition, there could be more comorbidities in ischemic patients, as they are generally relating to atherosclerosis in the whole body. A subanalysis of the MADIT II demonstrated reduced or lack of ICD benefit in patients with the highest comorbidities.^17^ This evidence may have precluded us from implanting CRT‐D and ICD in ischemic patients ≧75 years with high comorbidities.

Based on the analysis of the JCDTR, determinants for selecting primary prevention CRT‐D over CRT‐P in patients with symptomatic HFrEF were younger age, male, reduced LVEF, a history of nonsustained ventricular tachycardia (NSVT).^7^ As expected, there was a remarkable disparity of age groups in ICD/CRT‐D/CRT‐P implanted patients, with the highest percentage of advancing age in CRT‐P recipients (Figure 2). However, we do not have an appropriate answer regarding choice of CRT devices, ie, CRT‐D or CRT‐P, for HFrEF patients with a QRS duration ≥130 m/s and left bundle branch block (LBBB) QRS morphology without prior sustained ventricular arrhythmias. This is because randomized controlled trials that directly compare the effects of CRT‐D and CRT‐P on morbidity and mortality in such heart failure patients are scarce.^2^, ^8^, ^9^

Several observational studies could not identify symptomatic heart failure patients who benefit more from CTR‐D than CRT‐P, as there were significant demographic and morbid differences between the two patient groups.^14^, ^18^, ^19^ Despite this, the superiority of CRT‐D to CRT‐P was reported in HFrEF patients with ischemic cardiomyopathy,^20^, ^21^, ^22^, ^23^ those with nonischemic cardiomyopathy having left ventricular midwall fibrosis^24^ and those with the Goldenberg (MADIT) risk scores 0‐2.^25^ More recently, CRT‐D was not associated with prolonged survival especially in nonischemic cardiomyopathy and no previous history of ventricular arrhythmias, as compared to CRT‐P.^26^, ^27^ Besides, in systolic heart failure patients aged ≧75 years^28^ or ≧80 years,^29^ there was no significant difference in the risk of mortality between CRT‐D and CRT‐P groups after adjusting for baseline differences. Since (a) CRT‐Ds are larger and more expensive than CRT‐Ps and (b) the predominant etiology of heart failure is nonischemic in Japan, we need to perform future research focusing on not only major cardiac events and mortality but also quality of life and cost effectiveness in symptomatic heart failure patients undergoing CRT‐D vs CRT‐P especially in advancing age of ≧75 years.

### 4.1 Study limitations

There are several limitations to be considered in this study. First, clinical outcomes are not shown in this study, but we reported several outcomes in other studies.^19^, ^30^ Second, the rate of registration with the JCDTR is decreasing as evident from the data of JADIA. For example, ratio of registration of the JCDTR to that of JADIA (JCDTR/JADIA ratio) was 57.0% for ICD and 49.3% for CRT‐D in 2011, but it declined gradually. In 2016, the JCDTR/JADIA ratio was 33.6% for ICD, 32.6% for CRT‐D and 21.1% for CRT‐P (Figure 1B). The second version of JCDTR (New JCDTR) is now operative (https://membnew.jhrs.or.jp/newjcdtr/) and data of the implantation and follow‐up in ICD/CRT‐D/CRT‐P implanted patients after January 2018 are prospectively enrolled. We hope the New JCDTR will be able to provide firm and further evidence of Japanese patients.

## 5 CONCLUSIONS

There has been an apparent increase in the percentage of de novo ICD/CRT‐D/CRT‐P implanted in patients aged ≧75 years in Japan. The implantation for primary prevention ICD and CRT‐D as well as in nonischemic cardiomyopathy contributed to the temporal increase in CIED implantation in advancing age.

## CONFLICTS OF INTEREST

The authors declare no conflict of interest related to this study.
